# Correlation Between Meso-Defect and Fatigue Life Through Representing Feature Analysis for 6061-T6 Aluminum Alloys

**DOI:** 10.3390/s26020631

**Published:** 2026-01-17

**Authors:** Liangxia Zhang, Yali Yang, Hao Chen, Shusheng Lv

**Affiliations:** 1 School of Mechanical Engineering, Ningxia Institute of Science and Technology, Shizuishan 753000, China; zlx0524@126.com; 2 School of Mechanical and Automotive Engineering, Shanghai University of Engineering Science, Shanghai 201620, China; carolyn71@163.com; 3 Chinese Academy of Agricultural Mechanization Sciences, Beijing 100083, China; lsscnq@163.com

**Keywords:** meso-defect, representing feature, relative weights, mesoscopic damage variable, fatigue life

## Abstract

Fatigue strength is vital for engineering applications of aluminum alloys. Accurate models incorporating mesoscopic defect-representing features are one of the issues for accurate fatigue strength prediction. A fatigue life prediction method based on meso-defect-representing features is proposed in this study. Based on staged fatigue damage, meso-defect data was obtained by X-ray CT. After 3D reconstruction and simplification, porosity, shape, and location were selected as the meso-defect-representing features using correlation coefficient analysis. Weights of meso-defect features were determined through FEM simulation. A mesoscopic damage variable incorporating the weights of porosity, shape, and location for meso-defect was defined. Correlation between fatigue life and meso-defect features was established through the mesoscopic damage variable. Experimental verification results showed that the prediction method is an effective method for fatigue life assessment.

## 1. Introduction

Aluminum alloy is widely used in aerospace and vehicle manufacturing due to its good casting properties and light weight. In the process of engineering application, a large number of accidents are related to the defects caused by the fatigue failure of materials. Defects are considered to be one of the main factors that seriously affect the mechanical properties and fatigue properties of materials [1,2,3,4,5].

For damage defect-based fatigue life prediction, efforts have been made to characterize material damage. One of the common approaches is the damage factor (D) based on continuous damage mechanics [6,7,8]; for non-destructive material, *D* = 1, and for complete loss of load-bearing capacity, *D* = 1. Chaboche proposed the concept of effective stress to establish a one-dimensional nonlinear damage accumulation model, in which the damage variable (D) is a function of the stress state and the current damage state [9]. Lemaitre proposed a fatigue damage evolution model based on cumulative plastic strain [10], which has been continuously improved, and different forms of models have been established for high- and low-cycle fatigue. Subsequent damage accumulation models are basically based on the damage evolution model of Chaboche and Lemaitre.

Meso-defects are the critical factors in determining the fatigue life of aluminum alloys. The characterization of meso-defect features, such as quantity, shape, size, and distribution [11,12], is critically important for understanding and predicting fatigue damage evolution [13]. Due to the enormous numbers, irregular shape, and uneven spatial distribution of meso-defects in the material domain, correlative research between defect features and fatigue damage behavior has been conducted, along with the development of continuum damage mechanics theory and high-resolution three-dimensional inspection technology (such as synchrotron radiation X-ray computed tomography) for the internal structure of materials [14,15,16]. Quantitative methods have been proposed for fatigue life prediction through defect population [17], porosity [18,19], size [20,21,22], defect shape [23,24] and location [25,26,27], influence of defect distance to the surface [28,29], combination of density, shape, and size [30], and so on, through numerical methods [31,32], the finite element method [33], and machine learning methods [34,35].

Due to the multi-scale characteristics of meso-defects and also their evolution during the fatigue process, it is vital to identify the contribution of different meso-defect-representing features to fatigue life. However, most studies are based on one or two characteristics, rather than on complex, heterogeneous meso-defects in real aluminum alloys. The critical research gap lies in moving from defect statistics to a physically graded understanding of fatigue damage initiation [36]. Tammas-Willliams et al. (2015) statistically correlated individual pore features with fatigue performance but did not combine them into a mechanistic damage variable [37]. There are few studies that combine these defect-representing features to characterize the degree of material fatigue damage.

Aiming to address the research gap in fatigue life prediction through an integrated framework for meso-defect features, a mesoscopic damage-representing feature-based fatigue strength evaluation method was proposed in this study. Our objective is to capture meso-defects that have developed sufficiently to significantly affect local mechanical responses and can serve as effective indicators of damage in order to establish a physically informed, mesoscale framework for quantifying the influence of meso-defects on fatigue life. After staged fatigue experiments, meso-defect data was obtained, reconstructed, and simplified. Meso-defect-representing features were selected and incorporated into a mesoscopic damage variable using a weighting method. A fatigue life prediction method based on the mesoscopic damage variable was proposed and experimentally verified, thus providing a relatively comprehensive method combing multiple meso-defect features for quantitative analysis for fatigue behavior.

## 2. Material and Methods

### 2.1. Material and Specimen

**Figure 1:**
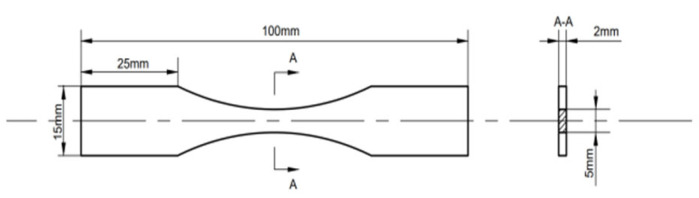
Dimensions of specimens.

The material of the test piece is 6061-T6 aluminum alloy. The chemical composition is shown in Table 1. The specimens are made in accordance with the requirements of ASTM E8/E8M-15a for fatigue testing. The model and dimensions of the specimens used are shown in Figure 1.

### 2.2. Staged Fatigue Damage Experiment

A tensile fatigue damage experiment was conducted using an MTS electro-hydraulic servo dynamic test system (model 370.10) at room temperature. The test adopted sinusoidal waveform control, and under the axial loading with a frequency of 50 Hz, the stress ratio was set to R = 0.1. Due to the variability of fatigue life testing, the final fracture point from direct physical measurement is not available for all calibration scenarios. A fixed fracture cycle of 100,000 cycles was defined as the rupture point, based on several pretesting, to provide a clear and unambiguous normalization point for comparing damage trajectories across different specimens and conditions in the dataset. Over the entire damage process from zero damage to rupture (100,000 cycles), 5 stages were selected—20,000 cycles, 40,000 cycles, 60,000 cycles, 80,000 cycles, and 100,000 cycles—to determine stage-wise damage behavior. For each stage, 3 replications were obtained.

### 2.3. Defect Data Acquisition

**Figure 2:**
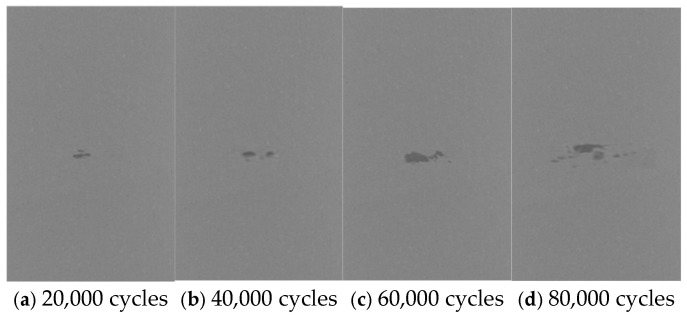
Tif diagrams for different loading stage specimens.

After the staged fatigue damage experiment, the center sections (6 × 5 × 2 mm^3^) of the specimens were scanned using an X-ray CT system (Pheonix v|tome|xm, GE, Tempe, AZ, USA) to obtain internal meso-defect information, with a resolution of 5 μm. For each specimen, non-destructive internal testing using the CT system scanned 1600 images over 360°. Figure 2 shows four typical Tif slices for different load stages.

**Figure 3:**
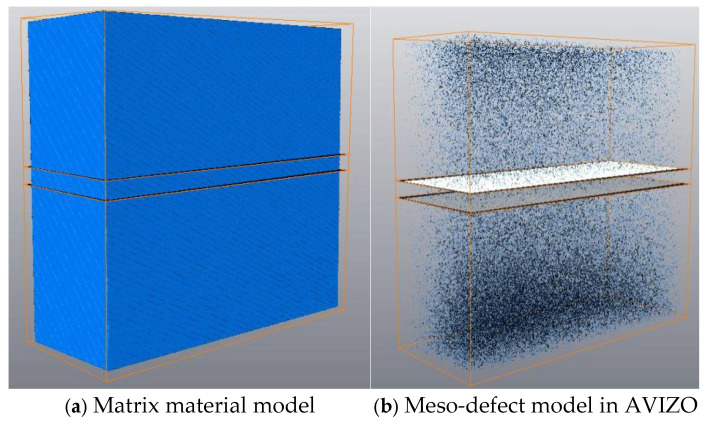
Visualization of meso-defects.

To obtain 3D information of meso-defect, AVIZO (2016) software was used to visualize and reconstruct damage data from X-ray CT. Two-dimensional image data were transferred into three-dimensional damage data. Defect data were reconstructed in three dimensions through positioning and display, filtering, defect segmentation, and recognition, as shown in Figure 3.

Then, damage parameters of specimens at different stages were obtained by the Label Analysis module in AVIZO. These parameters included damage characteristic information such as porosity, shape, size, and location of the defects.

### 2.4. Defect Shape Simplification

The shape of a defect can be defined by its sphericity and aspect ratio as representations of shape irregularity and elongation [38]. Generally, the detected defects had irregular shapes with sphericities much less than unity, and both sphericity and aspect ratio typically decrease with increasing defect size. Therefore, the aspect ratio of a defect is commonly used in fracture mechanics-based approaches for life prediction to represent the defect shape. In this study, a rotating ellipsoid was used to simplify defect shape. The spheroid is a geometric shape formed by rotating an ellipse 180° around its short axis. The defect volume V and surface area S were calculated to obtain the semi-major axis a and semi-minor axis b of the spheroid. The calculation formulas are given in Equations (1) and (2):

(1) $$V=\frac{4\pi ab^{2}}{3},$$

(2) $$S=\frac{4\pi \left(2ab+b^{2}\right)}{3}.$$

The shape of the ellipsoid can be expressed by the aspect ratio α, as expressed in Equation (3):

(3) $$\alpha =\frac{a}{b}.$$

**Figure 4:**
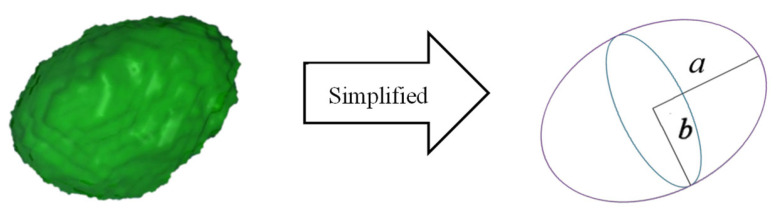
Simplification of defect shape.

The shape of the irregular defect was simplified into a regular ellipse, greatly improving the accuracy of defect recognition, as shown in Figure 4.

### 2.5. Weight Index Calculation

To identify the critical representing feature for meso-defects, the Pearson correlation coefficient was introduced [39], in which the Pearson correlation coefficient reflects the stability of relationships along with changes for variables through overall covariance calculation.

(4) $$\gamma_{xy}=\frac{\frac{\sum_{i=1}^{n}\left(x_{i}-\bar{x})(y_{i}-\bar{y})\right)}{n-1}}{S_{x}S_{y}\sqrt{\frac{\sum_{i=1}^{n}{\left(x_{i}-\bar{x}\right)}^{2})}{n-1}}\sqrt{\frac{\sum_{i=1}^{n}{\left(y_{i}-\bar{y}\right)}^{2})}{n-1}}},$$

where *x* and y represent two sets of data, $\left\{x_{1},x_{2},\cdots ,x_{n}\right\}$ and $\left\{y_{1},y_{2},\cdots ,y_{n}\right\}$. $S_{x}$ and $S_{y}$ are the standard deviations of the following data: $S_{x}=\sqrt{\frac{\sum_{i=1}^{n}{\left(x_{i}-\bar{x}\right)}^{2})}{n-1}}$, $S_{y}=\sqrt{\frac{\sum_{i=1}^{n}{\left(y_{i}-\bar{y}\right)}^{2})}{n-1}}$.

The relative weights of the chosen representing indices for meso-defect can be calculated as follows:

(5) $$\omega_{i}=\frac{k_{i}}{\sum_{n=1}^{3}k_{n}},$$

where $\omega_{i}$ is the weight of the defect feature $S_{i}$ relative to the other defect features.

### 2.6. FEM Model

For weight index calculation, the stress concentration factor was used as the main factor. The finite element method was implemented for stress concentration factor calculation along with meso-defect-representing index changes. An FEM model was established for the center section of the specimen in a 6 × 5 × 2 mm^3^ domain, the same as the CT scanning section. Meso-defect data after 3D reconstruction (Figure 3) was imported into ABAQUS. The characteristic information of the defects was imported through an ABAQUS subroutine.

**Figure 5:**
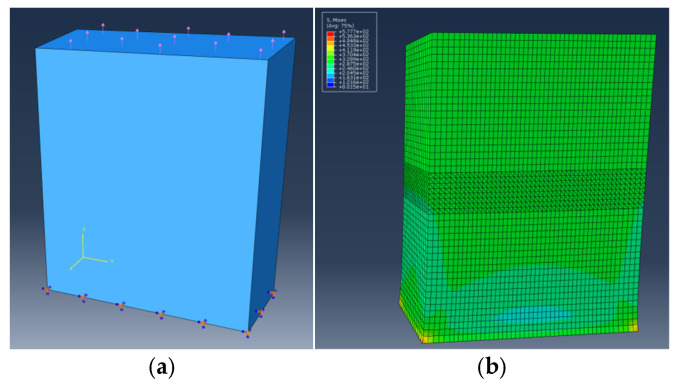
(**a**) Tension model; (**b**) simulation results.

The simulation was divided into 3 groups according to defect characteristics, and each group performed 5 simulation experiments by changing the characteristic quantities. The material was modeled as linearly elastic and homogeneous at the mesoscale, a standard assumption for studying the elastic stress concentration caused by isolated meso-defects prior to plasticity or damage evolution. A displacement control method was adopted to carry out the finite element tensile analysis of the damage model. One end of the model was fixed, and stress (p) was applied to the other end on the other end, as shown in Figure 5. From the final tensile simulation results, the value ($p_{\max}$) of the maximum stress point was obtained, as shown in Figure 5b. The stress concentration factor (k) was uniformly introduced as an intermediate variable for each group of simulations to determine the degree of material damage. The stress concentration factor (k) of the damage model can be obtained by Equation (6):

(6) $$k=\frac{p_{\max}}{p}.$$

The stress concentration factor was obtained through simulation using the above model. To establish a transparent and interpretable baseline model to preliminarily identify the main effects of different defect features on damage accumulation, a single-parameter perturbation method was used, in which the characteristic quantity of defect was increased by 10% each time. In order to avoid the mutual influence of defect spacing, when the porosity of defects increased, the defects size was increased proportionally while keeping the original defect positions unchanged. For the aspect ratio, the initial shape was assumed to be 1. For the position index, the volume of defects remained the same, but the distribution and arrangement of defects changed.

## 3. Results and Discussions

### 3.1. Meso-Defect-Representing Features

Table 2 presents the meso-defect-representing feature information at different fatigue stages, including number, porosity, aspect ratio, and position to the surface, after X-ray CT scanning, reconstruction, and simplification. Defects with volumes greater than 10^−8^ mm^3^ were taken into consideration for defect number. As meso-defects were simplified into ellipsoid, the representing index for shape was the average aspect ratio. The location-representing index was defined as the average distance to the surface. Defect number decreased with increasing load cycles, whereas porosity, aspect ratio, distance to the surface increased with load cycles.

### 3.2. Meso-Defect-Representing Feature Correlation Coefficient

**Figure 6:**
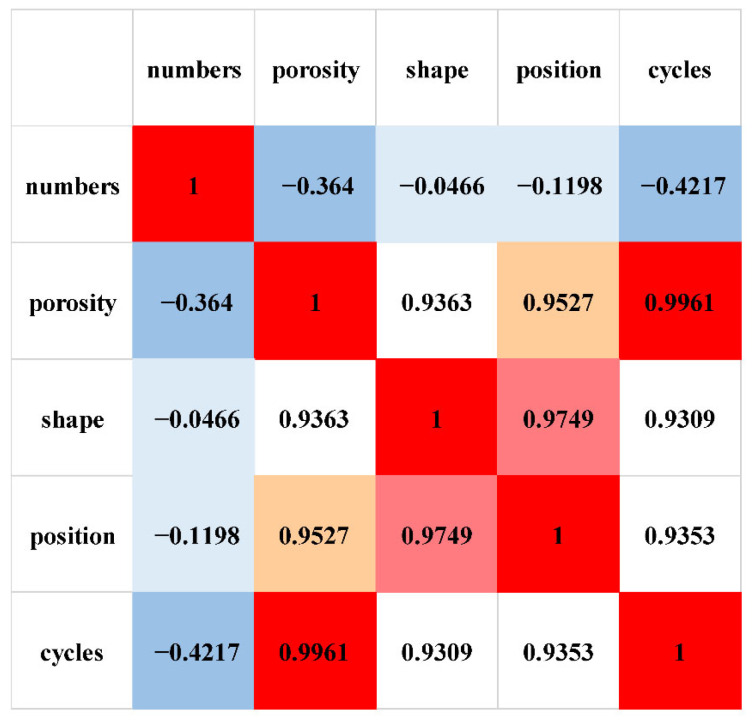
Correlation coefficient between representing features and fatigue cycles.

For Pearson correlation calculation, the fatigue cycle number was selected as an intermediate parameter, and the average values in Table 2 for defect number, porosity, aspect ratio (shape), and distance to the surface (position) were used for correlation analysis. As shown in Figure 6, a weak correlation was observed between meso-defect number and damage cycles. For porosity, shape, and position, strong correlations were observed, with coefficients greater than 90%. Therefore, three major meso-defect features—porosity, aspect ratio, and position to surface—were taken as the representing indices for further damage assessment.

### 3.3. Weight Index for Representing Features

**Figure 7:**
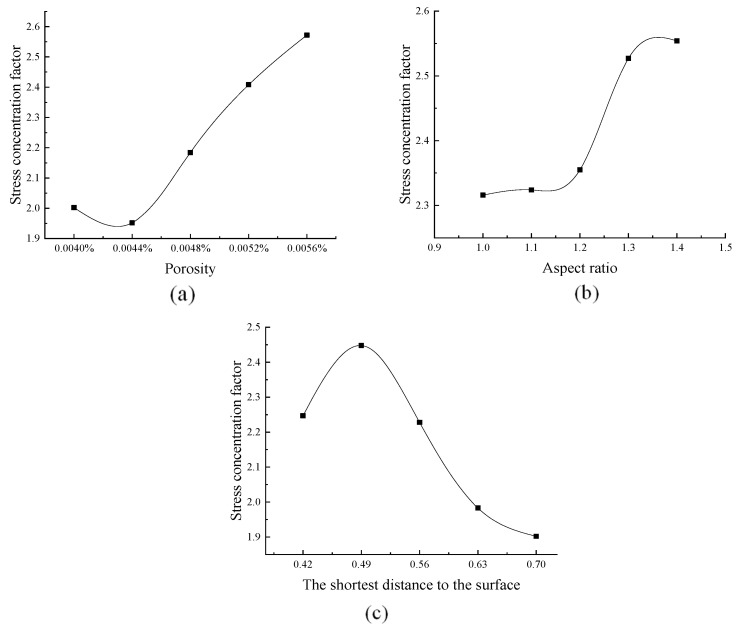
Change in stress concentration factor with defect feature, (**a**) porosity, (**b**) shape, and (**c**) position.

Based on the above FEM model, the stress concentration factor can be calculated, along with variations in porosity, aspect ratio, and shortest distance to the surface, as shown in Figure 7. The stress concentration factor value increased with changes in porosity and aspect ratio and decreased with decreasing distance to the surface.

These values can be fitted into a linear function using the least-squares method. The function can be described by Equation (7):

(7) $$\{\begin{array}{c} y_{1}=39900x_{1}+0.3080\\ y_{2}=0.6793x_{2}+1.5998\\ y_{3}=-1.649x_{3}+3.0851 \end{array},$$

where x_1_, x_2_, and x_3_ represent porosity, aspect ratio, and short distance to surface, respectively, and where y_1_, y_2_, and y_3_ represent the corresponding stress concentrate factors.

Based on the previous hypothesis, the defect characteristic variable $x_{i}$ increases by 10% and the mesoscopic damage variable $x_{g}$ will correspondingly increase by $k_{i}\%$. The elastic coefficients of the defect characteristic indices for porosity, shape, and location can be calculated as k_1_ = 8.38, k_2_ = 2.98, and k_3_ = 5.98. Thus, the relative weights of porosity, shape, and location can be obtained by the above-mentioned weight calculation method Equation (5) as $\omega_{1}=0.483,\omega_{2}=0.172$, and $\omega_{3}=0.345$, respectively.

### 3.4. Mesoscopic Damage Variable

The mesoscopic damage variable is fundamental for formulating constitutive models that describe the progressive failure process in engineering materials [40]. Due to the uneven size and distribution of meso-defects, a comprehensive damage variable was proposed by combining porosity, shape, and location to represent complex, heterogeneous meso-defects in aluminum alloys.

For accurately describing material failure under the fatigue process, incorporating meso-defect feature indices—porosity, shape, and location—into a comprehensive index is essential. This index, the mesoscopic variable ***d***, was obtained from the relative weights of the representing features, as shown in Equation (8). The mesoscopic variable ***d*** varies within the range [0, 1]. At the rupture point (100,000 cycles), ***d*** = 1.

(8) $$d=\omega_{1}\frac{P_{i}}{P_{m}}+\omega_{2}\frac{S_{i}}{S_{m}}+\omega_{3}\frac{L_{i}}{L_{m}},$$

where $\omega_{1},\;\omega_{2},\;and\;\omega_{3}$ are the relative weights corresponding to porosity, shape, and position of defects. $P_{i},\;S_{i},\;{and\;L}_{i}$ are the damage equivalents of the three features at a given cycle stage (porosity, shape, and location), as shown in Table 2. $P_{m},S_{m},\;and\;L_{m}$ are characteristic quantities corresponding to complete damage in terms of porosity, shape, and location.

To verify the effectiveness of the mesoscopic damage variable, the macroscopic damage variable ***D*** was introduced for validation of the defined mesoscopic damage variable.

(9) $$D=\frac{k_{e}-k_{min}}{k_{max}{-k}_{min}},$$

where $k_{e}$ is the stress concentration factor corresponding to the equivalent damage of the material, $k_{\min}$ is the stress concentration factor the undamaged material, and $k_{max}$ is the stress concentration factor corresponding to the maximum damage of the material. As the maximum fatigue life of the test specimen obtained from preliminary experiments was approximately 100,000 cycles, the stress concentration factor at the 100,000-cycle stage was defined as the maximum stress concentration factor.

The relative error between the mesoscopic and macroscopic variables can be calculated, and then the average value can then be used as the similarity δ, as shown in Equation (10):

(10) $$\delta =\sum_{i=1}^{5}\frac{\left|d_{i}-D_{i}\right|}{D_{i}}\times 100\%.$$

By substituting the relative weights and representing index data for different fatigue stages in Table 2 into Equation (8), the mesoscopic damage variable *d* and the macroscopic damage variable ***D*** can be obtained, as shown in Table 3.

A quite similar trend was observed for the mesoscopic variable ***d*** and the macroscopic damage variable ***D***, increasing with load cycles from 0 to 1, which is similar to the normal damage variables reported in previous studies [7,41]. The biggest difference was observed at the 20,000-cycle stage, probably because of the influence of more than 30,000 small meso-defects (Table 2). As load cycles increased, larger meso-defects developed, and the difference between ***d*** and ***D*** was reduced. The average relative error between the mesoscopic variable ***d*** and the macroscopic variable ***D*** is 6.9%, showing good agreement in damage evolution. Due to the fusion of 3 representing features (porosity, shape, and location) in the mesoscopic variable ***d***, the calculation conditions may be closer to the real fatigue conditions of aluminum alloys.

The reported average error of 6.9% is a global metric calculated across all stress amplitudes within the calibration dataset. This error is not constant and is expected to vary with stress amplitude due to underlying damage mechanisms. For broader application, this method can be utilized for both low-cycle and high-cycle fatigue. For low-cycle fatigue, the error may increase. Due to the linear-elastic matrix assumption, the macroscopic damage variable integrates significant plastic dissipation and micro-crack coalescence—nonlinear processes that are not explicitly captured by the current mesoscale model. For high-cycle fatigue, the error might be lower, as damage evolution is primarily elastic and the linearized, elasticity-based mesoscopic damage variable better approximates macroscopic stiffness degradation.

Furthermore, as normalization of the mesoscopic damage variable ***d*** was a key model assumption, fracture was enforced to occur at 100,000 cycles, and d was consequently normalized to unity. Instead of macroscopic elastic-modulus-based normalization, fracture was defined at a fixed observed cycle number (100,000 cycles), to provide a clear and unambiguous normalization point for comparing damage trajectories across different specimens and conditions in our dataset. This approach is common in continuum damage mechanics when the final fracture point from direct physical measurement is not available for all calibration scenarios. Modulus-based normalization will be included in future work. A plan to measure the critical elastic modulus drop at fracture in subsequent experiments will be outlined to refine the model’s terminal boundary condition.

### 3.5. Fatigue Life Assessment Based on Mesoscopic Damage Variable

**Figure 8:**
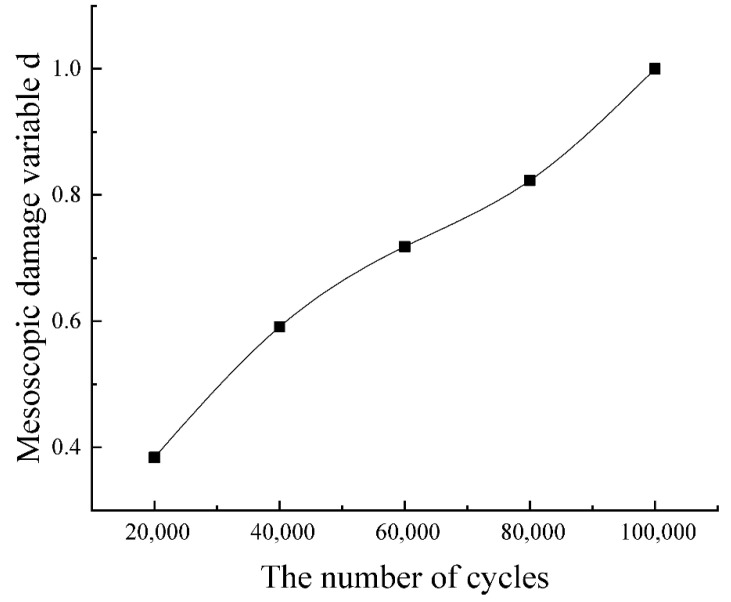
Mesoscopic damage variable in different fatigue stages.

The mesoscopic damage variable serves as a critical bridge between microstructural degradation and macroscopic fatigue failure. The mesoscopic damage variable ***d*** in Table 4 can be derived separately, as shown in Figure 8. The mesoscopic damage variable ***d*** increases with loading cycles from 0 to 1 until fracture. This result is similar to previous studies of damage variable D based on Young’s modulus degradation [21] and the multiscale fatigue damage index (MDFI) incorporating meso-scale defects [42], demonstrating the effectiveness of the proposed mesoscopic damage variable.

By polynomial regression, the relation between fatigue life (N) and mesoscopic damage (***d***) can be obtained, as shown in Equation (11):

(11) $$N=-64.572d^{4}+129.08d^{3}-72.008d^{2}+17.504d+0.0004.$$

For continuum damage mechanics, the uncertainty of constitutive relationships tends to increase as they approach the boundaries of the calibration range. To ensure the prediction capability of the proposed model, the validation point was chosen within the experimental testing cycle range [0, 100,000]. For verification, the fatigue stage at 50,000 cycles was selected as a separate fatigue stage.

The fatigue test was carried out using the same experimental parameters settings, with three replications. For comparison, the same procedures were conducted, including fatigue testing, CT scanning, 3D reconstruction, simplification, and so on. Porosity was obtained through AVIZO. For shape, the aspect ratio was calculated after simplification. For location, the shortest distance from the meso-defect coordinate position to the surface was used. Porosity, shape, and location at 50,000 cycles are listed in Table 4.

**Table 4:** Representing features of meso-defects under 50,000 fatigue cycles.

Meso-Defect Representing Feature	Porosity	Shape	Location
Parameter	0.189	0.834	0.913

Thus, the mesoscopic damage ***d*** for 50,000 cycles was 0.648 from Equation (8). Based on Equation (10), the predicted fatigue life was 48,436 cycles. The error between the prediction method and the actual test was 3.13%, indicating relatively high accuracy. This illustrates that the model’s predictions within the primary range of interest are accurate and physically consistent. Therefore, the proposed mesoscopic damage ***d***, incorporating meso-defect porosity, shape, and location, can be used for fatigue life assessment.

In the current study, we primarily focused on validating the model’s interpolation performance within the fitted range, which forms the foundation of the model’s reliability. A physically informed mesoscale framework for quantifying defect influence was established. The model relies on linear superposition of scalar damage contributions from meso-defects features, thereby neglecting nonlinear interactions. The study is currently based on 6061-T6 aluminum alloy under constant-amplitude loading to establish a proof of concept and calibrate the core defect-weighting methodology. The model reliably predicts fatigue life for meso-defect features and stress levels within the calibrated domain. For other alloys, arbitrary meso-defect distributions, or different production techniques, the baseline model required re-calibration of material-specific parameters for different materials and loading spectra. The generalizability of the proposed model should be further enhanced. Future extrapolation tests are an essential step in comprehensively assessing the model’s robustness.

## 4. Conclusions

Based on staged fatigue experiments, meso-defect features were obtained and processed. A mesoscopic damage variable d incorporating defect porosity, shape, and location was established, along with a corresponding fatigue life prediction method. The following conclusions can be drawn.

(1) Based on simplified meso-defects, porosity, shape, and location were selected as the meso-defect-representing indices using correlation coefficient analysis.
(2) The weights of meso-defect-representing features were determined through FEM simulation based on stress concentration factor calculations.
(3) A mesoscopic damage variable ***d*** was determined using the weight method, along with a macroscopic damage variable D derived from the stress concentration factor. The average relative error value between the mesoscopic damage variable ***d*** and the macroscopic damage variable ***D*** was 6.9%.
(4) The relationship model between the mesoscopic damage variable ***d*** and fatigue life is established. Verification using the 50,000-cycle experiment showed an error of 3.13% between the proposed prediction method and the experimental result, validating the effectiveness of this method.

Further exploration of this baseline model should focus on the quantitative interaction relationships among meso-defect-representing features and on model normalization to better elucidate the mechanisms by which meso-defect influence fatigue damage evolution and to enable more accurate fatigue life assessment.

## Figures and Tables

**Table 1:** Chemical composition of 6061-T6 alloy used in this study (wt.%).

Al	Mg	Fe	Si	Cr	Cu	Zn	Mn	Ti	Other
95.8	0.8~1.2	0.7	0.6	0.3	0.2~0.4	0.25	0.15	0.15	0.15

**Table 2:** Simplified defect information of staged fatigue test.

Load Cycle (×10^3^)		20	40	60	80	100
Total number of defects	Group 1	35,106	215,398	125,703	35,684	20,982
	Group 2	36,061	233,821	104,895	30,569	16,456
	Group 3	34,854	190,636	76,695	24,534	18,672
Porosity (%)	Group 1	0.035	0.166	0.268	0.338	0.343
	Group 2	0.076	0.141	0.219	0.288	0.376
	Group 3	0.06	0.176	0.242	0.343	0.439
Aspect ratio	Group 1	0.434	0.659	0.783	0.859	0.887
	Group 2	0.329	0.614	0.681	0.731	0.739
	Group 3	0.404	0.713	0.787	0.798	0.855
Average distance to the surface (mm)	Group 1	0.894	0.852	1.137	1.061	1.233
	Group 2	0.92	1.038	1.204	1.15	1.412
	Group 3	1.045	1.512	1.334	1.435	1.314

**Table 3:** Macroscopic damage and mesoscopic damage at each stage.

Cycle (×10^3^)	20	40	60	80	100
*D*	0.464	0.617	0.783	0.865	1
*d*	0.384	0.591	0.718	0.823	1
δ	17.2%	4.2%	8.3%	4.9%	0

## Data Availability

The data presented in this study are available on request from the corresponding author. The data are not publicly available due to technical confidentiality.
